# Iron oxide/graphene oxide nanocomposite synthesis using atmospheric cold plasma†

**DOI:** 10.1039/d3ra05560d

**Published:** 2024-01-08

**Authors:** Andjelika Bjelajac, Adrian-Marie Phillipe, Jérôme Guillot, Jean-Baptiste Chemin, Patrick Choquet, Simon Bulou

**Affiliations:** a Luxembourg Institute of Science and Technology 28, Avenue des Hauts-Fourneaux L-4365 Esch-sur-Alzette Luxembourg andjelika.bjelajac@list.lu

## Abstract

Herein, we demonstrate the use of an atmospheric pressure plasma with a Dielectric Barrier Discharge (DBD) for the synthesis of FeO_*x*_ nanoparticles with a simultaneous formation of graphene oxide domains at low substrate temperature. For that, the interaction of the plasma to control good decomposition of the Fe precursor is essential and this is demonstrated by FTIR analyses. Thanks to a fine tuning of the plasma conditions, a homogeneous spatial distribution around 5 nm nanoparticles (NPs) was obtained, whereas without plasma, in the same configuration of the process, a heterogeneity regarding size and shape for the NPs was obtained. The Raman spectrum of the plasma deposit confirmed the presence of graphene oxide as the characteristic G and D bands were observed with *I*(D)/*I*(G) = 0.92. Thanks to optical emission spectroscopy (OES) measurements, it is proposed that the carbon deposition on FeO_*x*_ nanoparticles is produced on the near plasma post discharge. XPS studies showed that the main contribution of iron was in Fe^2+^ form, corresponding to the FeO phase. No metallic Fe or carbide were detected. As there are many studies reporting the synergetic effect of FeO_*x*_ NPs and graphene oxide, we believe that this new one-step simultaneous synthesis method may be of high interest for applications requiring direct deposition on temperature labile substrates such as polymers.

## Abstract 2

Atmospheric pressure cold DBD plasma for simultaneous synthesis of FeO_*x*_ nanoparticles and their partial graphenic encapsulation using ethanol as carbon source.

## Graphical abstract

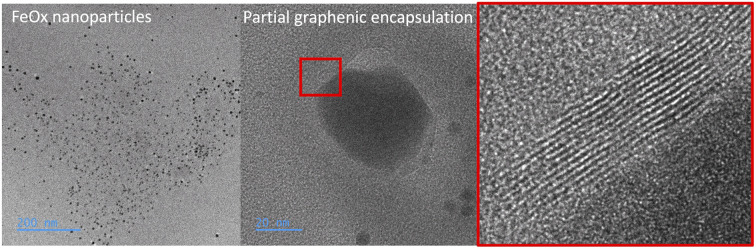

## Introduction

Nanoparticles (NPs) technology is a widely explored domain because the NPs' numerous applications depend on their properties: size, shape, defect density, and composition. For instance, Iron oxide (FeO_*x*_) NPs have attracted much attention in biomedicine,^1^ agriculture,^2^ and the environment,^3,4^ due to their low toxicity, superparamagnetic properties, and their simple separation methodology.^5^ To improve the stability of the FeO_*x*_ NPs as well as to enhance the materials performances, creation of nanocomposites containing FeO_*x*_ NPs is seen as advantageous. Many recent studies reported the beneficial role of graphene and its derivatives as graphene oxide (GO) incorporated as a FeO_*x*_ support for electrodes of water electrolyzers,^6^ Li- and K-ion batteries,^7^ supercapacitors,^8,9^ water purification systems (*i.e.* Cr(vi)^10^ or dye^11^ removal) or even magnetic resonance imaging,^11^ and cancer treatment. For some applications, advantage is given to GO with regards to graphene, as the presence of oxygen-containing functional and reactive groups (*e.g.*, carboxyl, epoxide, and hydroxyl) promotes wider applications, such as polymer composites, energy materials, sensors, transistors, and biomedical related applications. This is because of the great potential for surface functionalization and modification.^12–14^

GO is usually synthesized by exfoliating the bulk graphite, either mechanically or chemically, followed by the deposition on a designated substrate using chemical vapor deposition (CVD) on the metal catalyst and the Hummer's technique.^15^ CVD for GO synthesis is not only tricky and costly but due to the generation of uncontrollable yield and higher energy demands this method is being forsaken.^16^ The Hummer's process has been criticized due to the release of toxic gases like NO_2_, N_2_O_4_, and ClO_2_, which are explosive in nature. In contrast to these conventional fabrication procedures of GO requiring low pressure and high-temperature,^9^ plasma assisted methods reduce the operation temperature and they can be done at atmospheric pressure, like proposed by Alam *et al.*^17^ where plasma was generated with argon gas and methane was used as a carbon source. Herein, we propose the use of an atmospheric pressure (AP) dielectric barrier discharge (DBD) cold plasma torch method, and ethanol as a carbon source which is advantageous considering that ethanol can be obtained from the fermentation of agricultural industries.

Having in mind that DBD plasma was already proven as an effective method for NPs synthesis as well,^18^ we now explored the possibility of a simultaneous synthesis of FeO_*x*_ NPs and GO.^19^

The great majority of methods for preparation of FeO_*x*_ on graphene/GO involve multi-steps with prior synthesis of graphene/GO and subsequent FeO_*x*_ NPs decoration (either by covalent linkage or physical adsorption).^20,21^ Most of the time, the surface functionalization of graphene/GO is required to provide chemical groups for attaching FeO_*x*_ NPs. These multistep synthesis procedures are often time-consuming and difficult to control, resulting in hybrids with random and inhomogeneous coverage of graphene and/or GO surface by FeO_*x*_ NPs.^22^ Additionally, in these reactions, a significant fraction of graphene sheets is not coated with FeO_*x*_ NPs as graphene sheets are stacked together due to van der Waals interactions. Thus, one-step synthesis processes for graphene surface activation and FeO_*x*_ NPs grafting are favoured as both nanocomposite components are formed *in situ*. This way, the reaction can more easily be optimized (*e.g.*, the size of nanoparticles, the degree and uniformity of nanoparticles distribution over the graphene surface), and the physicochemical properties of the hybrids can be readily tuned with regards to the specific application requirements.^23^

Guo *et al.*^24^ reported the *in situ* graphitization catalysed by Fe_3_C/Fe. This study was based on the fact that iron forms a rather stable carbide but graphite precipitation from Fe can only occur under very specific conditions.^25^ For instance, the drawback of their method is that it required multi-steps as well as temperature higher than 800 °C. Furthermore, Sergiienko *et al.* reported the synthesis of Fe_3_C NPs^26^ using electric plasma discharge in liquid (such as ethanol) with graphitic carbon encapsulation. However, in that case the appropriate handling of the product had to be done, like filtration and waste treatment. The advantage of the method proposed herein, AP DBD plasma, is that is considered solvent-less and safe, without any toxic and dangerous side-products.

This study aims to investigate the influence of plasma on “in-flight” formation of FeO_*x*_ NPs from the aerosolized ethanol-based Fe precursor solution. We will demonstrate the benefit of using ethanol as solvent with regards to water, as it was recently reported for gold NPs encapsulation in carbon-based matrix preventing them from agglomeration.^19^

## Experimental

### Synthesis of FeO_*x*_@GO films

The experimental setup used here consisted of a vertically placed AP torch with a coaxial DBD geometry, composed of 2 concentrical hollow quartz tubes. The plasma was created between the tubes, where the inner tube was coated with Pt by a Physical Vapor Deposition (PVD) technique and the outer tube with Al foil. To produce the plasma, 10 slm of Ar are sent in the gap between the 2 tubes, and a sinusoidal HV is applied to the outer electrode (AFS generator, 52 kHz, 20 W) while the inner tube is connected to the ground. The exact details of the setup can be found in our previous publication.^19^ As Fe precursor of the NPs, iron(iii) acetylacetonate (Fe(acac)_3_, 97%, Sigma Aldrich) was dissolved in ethanol or water (concentration of 0.21 g l^−1^). The injection of 100 μl min^−1^ was done using a Hamilton 10 ml syringe and a syringe pump system. The microdroplets were produced thanks to an ultrasonic nebulizer (Sono-Tek®, 1 W, *f* = 120 kHz). Ar was used as carrier gas (10 slm flow rate) to carry the aerosol into the plasma near post-discharge. The prior optimization of the synthesis parameters was done to ensure the best outcome regarding the dispersion uniformity and NPs size distribution. Our previous study about Au NPs obtained using the same approach demonstrated the benefit of using plasma with these synthesis parameters.^19^ The distance between the plasma torch and the substrate was fixed at 10 mm, and the substrate temperature did not exceed 50 °C, whatever the deposition duration. Herein, we compared the deposits obtained with and without applied plasma (using nebuliser and the torch under the same deposition conditions).

**Fig. 1:**
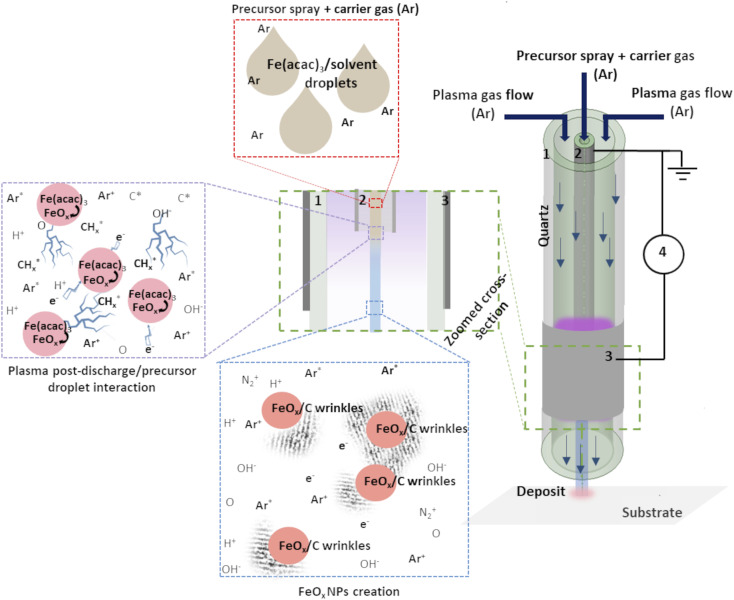
Schema of the experimental set-up: (1) outer quartz tube, (2) inner injection tube with Pt coating, (3) Al foil and (4) generator. Adapted from ref. 19.

The schema of the experimental setup is given in Fig. 1. To provide the insight of possible mechanism of FeO_*x*_ NPs creation within the setup, an assumed scenario is also presented.

Various substrates were used for the deposition according to the characterization technique to be carried out, *i.e.*, Si wafer for scanning electron microscopy (SEM/EDS) and X-ray photoelectron spectrometry (XPS), a 300 mesh Cu holey carbon grid for transmission electron microscopy (TEM), and a silica glass plate for optical measurements.

### Characterization techniques

The TEM analyses were done on a JEOL JEM-F200 cold FEG microscope operating at an acceleration voltage of 200 kV. Crystalline nanostructures were analysed by direct spacing measurements on High-Resolution TEM (HRTEM) images using Digital Micrograph Software from Gatan (version v.3.50.3584.0).

Laser Raman spectroscopy (inVia, Renishaw) was used at a wavelength of 532 nm with the power around 2.6 mW on a spot of 1 μm^2^, in order to differentiate between no-plasma and *in situ* plasma deposit.

X-ray photoelectron spectra (XPS) were acquired using a ThermoFisher Scientific Nexsa-G2 photoelectron spectrometer with a monochromatic Al Kα source (10 mA, 12 kV) and a 400 μm spot size. Fe 2p, C 1s, O 1s, and N 1s narrow scans were collected with an energy resolution of 0.6 eV, determined on a clean Ag foil. The spectra were reconstructed using Gaussian–Lorentzian peaks after removing a Shirley type background.

Optical Emission Spectroscopy (OES) was achieved using an Acton Series SP-2500i (Princeton Instruments) with 300 mm^−1^ grating (blaze wavelength 300 nm). A UV-vis optical fiber pointing at the lower edge of the plasma torch collected the photons emitted by the plasma discharge/aerosol interaction.

Fourier Transform Infrared (FTIR) characterisation of the nanocomposites deposited on Si wafer were performed using a BRUKER VERTEX 70FTIR in transmission mode.

The optical measurements were performed using LAMBDA 1050 UV-vis-NIR spectrophotometer from PerkinElmer with a 100 mm integration sphere. Measurements were performed in the UV-vis spectral range (300–1500 nm).

## Results and discussion

**Fig. 2:**
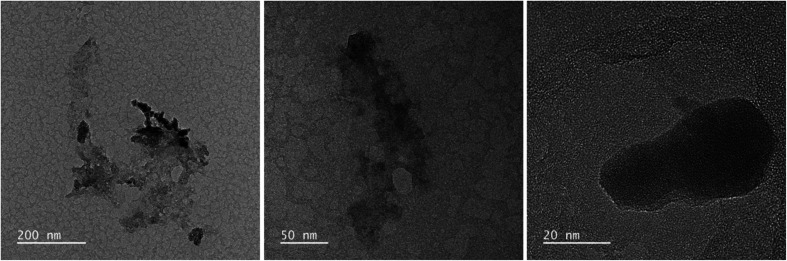
TEM micrographs overview of the deposit obtained using 0.21 g l^−1^ Fe(acac)_3_ in ethanol, 20 min without plasma.

**Fig. 3:**
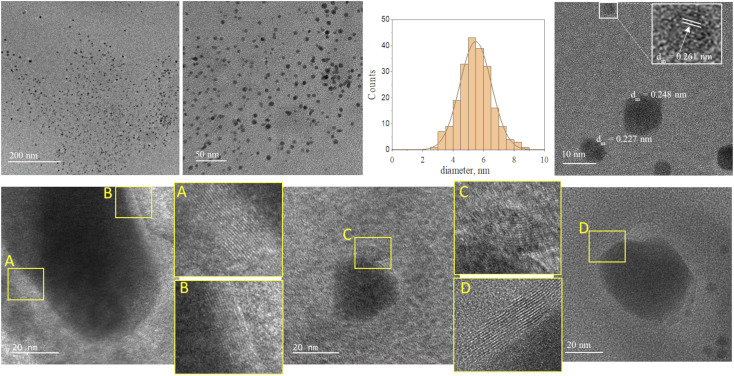
TEM micrographs overview of the deposit obtained using 0.21 g l^−1^ Fe(acac)_3_ in ethanol, 20 min with plasma.

**Fig. 4:**
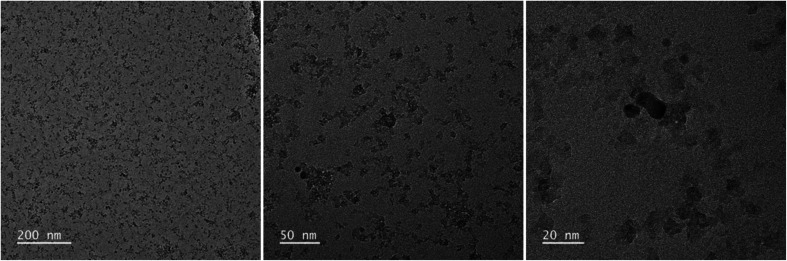
TEM micrographs overview of the deposit obtained using 0.21 g l^−1^ Fe(acac)_3_ in H_2_O, 20 min with plasma.

Fig. 2 and 3 provide the overview of TEM micrographs of the deposit obtained without and with plasma, respectively. The depositions were done directly on a Cu/holey C TEM grid. The no-plasma sample exhibits heterogeneous NPs population, both concerning the size and shape. The residual of Fe precursor was present throughout and there were some crystalline domains scarcely. With a plasma assisted deposition, NPs are found to be spherical and of narrow size distribution (220 counts, mean 5.4 nm, std dev 1.1). The presence of Fe, O and C was detected by EDS analysis (ESI Fig. S1†). However, the phase composition was not identified as the measured interplanar distances did not correspond to only one of the FeO_*x*_C_*y*_ family. The deposition was mainly uniform, and NPs were well isolated since no agglomerates were observed. However, NPs of ∼20 nm of irregular shape with graphitic layers (*d* = 0.34 nm corresponding to graphene (002), zoom areas A–D) nearby or even attached to the surface were observed. These graphitic layers (wrinkles) were not found in the deposit obtained without plasma. The source of C that creates the graphitic layers during *in situ* plasma deposition must be ethanol used as solvent, since using aqueous based Fe precursor there was no evidence of graphitization (Fig. 4) What is more, majority of the obtained deposit still contained Fe(acac), indicating a poor FeO_*x*_ NP germination rate. Whereas, ethanol-based aerosolized precursor enabled the creation of well dispersed ∼5 nm FeO_*x*_ spherical NPs and partial graphitization seen as wrinkles around NPs of ∼20 nm in size.

**Fig. 5:**
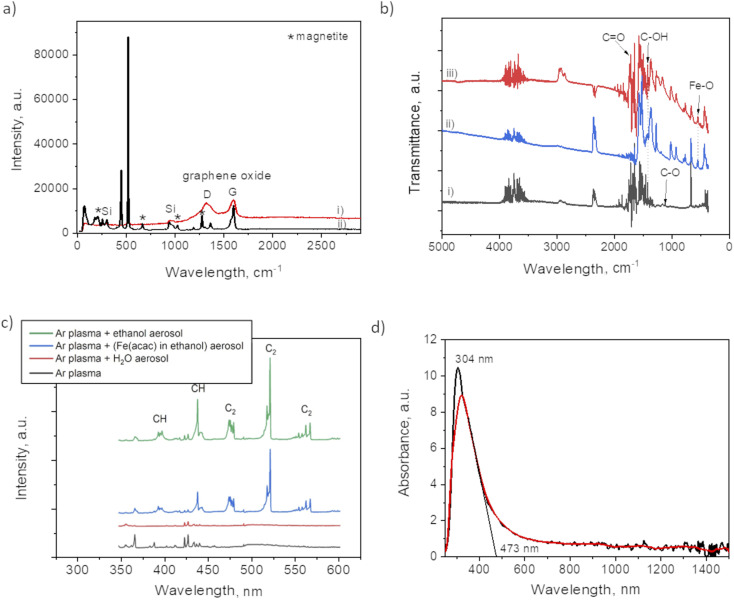
(a) Raman spectrum of: (i) plasma deposit and (ii) no-plasma deposit; (b) FTIR spectra of: (i) plasma deposit, (ii) the film obtained from drying the ethanol based precursor and (iii) no-plasma deposit; (c) OES spectrum of Ar plasma discharges with various aerosolized mixtures and (d) absorbance spectrum of film obtained after 90 min of plasma deposition.

Raman analysis was performed to assess the presence of graphitic domains on both samples, with and without plasma. For the plasma assisted sample (i) in certain domains, the Raman spectrum (Fig. 5a) shows the GO characteristic D and G band at 1353 cm^−1^ and 1605 cm^−1^, respectively. The absence of 2D at 2690 cm^−1^, reveals the lack of graphene within the nanocomposites thin films. Large width of the “D” peak may be considered as a confirmation of the presence of a variety of short graphitic fragments. An overlapping of the signals from the fragments of a different size results in the peak broadening.^27^ The intensity ratio for *I*(D)/*I*(G) was 0.92 which, also coincides with GO structure.^17^ The spectrum for the no-plasma assisted sample (ii) showed the characteristic peaks for magnetite at 210, 667, 1274 (marked by *).^28,29^ There was evidence of Fe(acac)_3_ residual, *i.e.* at 446 cm^−1^, assigned to a symmetric stretch of the ligands relative to the Fe atoms (Fe–O stretch and C–CH–C bend). O–Fe–O rocking motions were visible as band at 173 cm^−1^, and (O–Fe–; C–CH–C; O=C–CH_3_) bend at 255 cm^−1^. The band at 1600 cm^−1^ can be associated with the stretching *υ*(C–O) of the (acac) ligands confirmed by the presence of their most pronounced skeletal vibration at 1365 cm^−1^.^30^

The difference between the two samples preparation is also evident from FTIR spectra (Fig. 5b). The plasma sample (i) showed a peak at 1100 cm^−1^ that was not detected in case of no-plasma sample (iii). This peak was previously associated with the stretching of C–O in graphene.^31^ The no-plasma sample and the film obtained from drying the ethanol based precursor directly on Si wafer (ii) showed several characteristic bands in 730–1320 cm^−1^ (ref. 32 and 33) and a peak at 545 cm^−1^ that can be ascribed to Fe–O stretching mode.^34^

To get an insight of the reactive species appearing in the plasma post-discharge the OES analyses were performed. Indeed, by comparing the spectra (Fig. 5c) one can observe the emission of C_2_ (Swan system) and CH when ethanol aerosol was used for ethanol-mist, whereas these species were not detected when using water as a solvent. As CH and C_2_ species are known to be major precursors of graphene-based structures in CVD and PECVD processes,^35^ these results are a clear indication of the decomposition of ethanol due to its interaction with Ar plasma post-discharge resulting into partial graphitization of FeO_*x*_ NPs created *in situ*.

To investigate the optical response of the sample, we measured the absorbance of the film obtained after 90 min of plasma deposition. One can observe from the spectrum provided in Fig. 5d a peak at 304 nm with the absorption shoulder ending at 473 nm. Similar findings were reported by Niraimathee *et al.*^36^ indicating a surface plasmon resonance (SPR) of such small FeO_*x*_ NPs. The graphenic domains were reported not to influence the absorption properties of FeO_*x*_ NPs.^37^

**Fig. 6:**
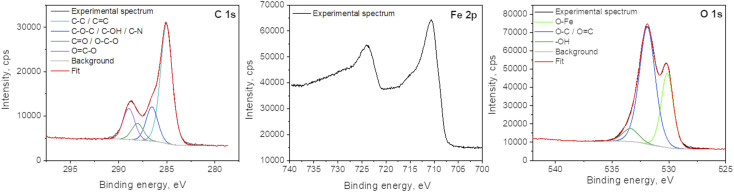
XPS spectra of C 1s, Fe 2p and O 1s of the plasma deposit.

X-ray photoelectron spectroscopy analyses were performed to determine the surface elemental composition of the plasma deposit sample (C = 43.1 at%; O = 38.2 at%; Fe = 16.3 at%; N = 1.2 at% and Cl < 0.5 at%). The slight N incorporation is likely to be due to the interaction with air, as the synthesis process is achieved in an open atmospheric environment. The various functional groups in GO were also investigated and particularly the chemical state of Fe in the FeO_*x*_ compounds (Fig. 6). The main C 1s component at 285.0 eV corresponds to C–C and C=C bonds.^38^ The other contributions are due to the partial oxidation of carbon atoms in the material (C–O–C and C–OH functions at 286.5 eV; O–C–O and C=O functions at 288.0 eV; O=C–O functions at 288.9 eV). One can note that iron carbide is not present in the sample as the peak characteristic of this phase, around 283.3 eV, is not observed in the C 1s spectrum. Some authors concluded in a previous work that the bonds between FeO_*x*_ and GO should be Fe–O–C.^31^ The Fe 2p_3/2_ spectrum is located at 710.5 eV and its associated satellite is observed approximatively 5.5 eV higher in the binding energies. The peak shape and the satellite energy gap are characteristic of Fe^2+^ as in FeO.^39,40^ However, the position of the peak is slightly shifter upwards. This can be due to the charging effect of FeO NPs being encapsulated in C matrix.^19,41^ Furthermore, one can note that no Fe^0^ contribution (sharp peak around 706.7 eV) and corresponding metallic iron nor iron carbide are observed in the spectrum. The two main contributions of the O 1s spectrum located at 531.9 eV and 530.2 eV attributed to CO_*x*_ compounds and Fe–O bonds, respectively.

Our findings shed the light of plasma post discharge/ethanol-based droplets carrying the precursor for FeO_*x*_ NPs creation. The DBD plasma generated species with high kinetic energies (up to several eV (ref. 42)) caused the decomposition of ethanol and the creation of C_2_ radicals providing graphitization around FeO_*x*_ NPs. Stancampiano *et al.* explained that this remarkable chemical reactivity of droplets in contact with plasma rises from the large surface-to-volume ratio of droplets and the plasma reactive species being in close proximity to the droplets' surface enhancing the transfer of activation energy from the plasma to the droplets. In this DBD plasma torch configuration droplets act as individual microreactors with controllable reactivity, enabling a range of conditions that cannot otherwise be achieved in batch processes.^43^ The biggest advantage of the presented process lies in the temperature that is far lower than the so-far reported *in situ* graphitization of Fe-based NPs. Most of the conventional processes, like the hydrothermal proposed by Guo *et al.*^24^ required multiple steps to obtain similar Fe@graphene nanohybrid at the temperatures higher than 800 °C. Plasma-assisted processes have emerged as a promising and complementary/alternative technology to conventional methods offering low synthesis temperature for the simultaneous synthesis of metal-oxide NPs with graphene-based domains. Dias *et al.* used manganese dioxide (MnO_2_) macroparticles that were dispersed in a hot zone of plasma created with a magnetron of 2.45 GHz and 2 kW microwave power.^44^ However, the DBD plasma torch, proposed here, operates at 20 W and 50 kHz using an AFS generator, does not require a water-cooled circulator and enables the deposition at temperatures lower than 50 °C. The estimation of the post-discharge gas temperature was done by measuring the rotational temperature of OH (*λ* = 306 to *λ* = 310 nm) from the OES data.^45^ This temperature is competitively lower than the current lowest temperature of graphene synthesis (>250 °C)^46^ achieved using atmospheric pressure microwave plasma.^47,48^ Therefore, we estimate the great perspective of this process as it opens a new horizon in low-temperature single-step preparation of metal-oxide@graphene-based nanocomposites.

## Conclusions

Herein, a one-step atmospheric pressure cold DBD plasma method was used for simultaneous synthesis of FeO_*x*_ NPs and FeO_*x*_@GO composite. An ethanol-based solution of Fe precursor in the form of an aerosol was carried to the post-discharge of plasma. Our findings showed that the plasma treatment was mandatory to decompose the Fe precursor and to obtain a homogeneous spatial distribution of the NPs. Plasma created FeO_*x*_ NPs present a good dispersity and narrow size distribution (mean diameter 5.4 nm), unlike scarce and low-quality deposit of heterogeneous size and shape obtained without plasma applied. Furthermore, we demonstrated that plasma had a double effect, apart from FeO_*x*_ NPs creation but also in their partial graphitic encapsulation. The OES analyses showed that when the ethanol-based Fe precursor was used a higher amount of CH and C_2_ species (known to be major precursors of graphene-based structures in CVD and PECVD processes) was detected pursuing the created FeO_*x*_ NPs. Raman spectrum of the plasma deposit confirmed the presence of graphene oxide as the characteristic G and D band were observed. XPS studies showed that the main contribution of iron was in Fe^2+^ form, corresponding to FeO phase. No metallic Fe or carbide were detected. As many studies report the synergetic effect of FeO_*x*_ NPs on GO, we believe that this new one-step simultaneous synthesis may be of high interest for applications requiring direct deposition on temperature labile substrate such as polymers. Herein, the substrate temperature did not exceed 50 °C, whatever the deposition duration.

## Author contributions

Andjelika Bjelajac: conceptualization, investigation, methodology; Adrian-Marie Phillipe: formal analysis, investigation; Jérôme Guillot: formal analysis, investigation; Jean-Baptiste Chemin: formal analysis; Patrick Choquet: funding acquisition, resources; Simon Bulou: funding acquisition, investigation, methodology, project administration, supervision, validation.

## Conflicts of interest

There are no conflicts to declare.

## Supplementary Material

RA-014-D3RA05560D-s001
