# Supplementary material for: Iron oxide/graphene oxide nanocomposite synthesis using atmospheric cold plasma
Source: RSC Adv. 2024 Jan 8;14(3):1750–6. doi: 10.1039/d3ra05560d (PMC10772707; doi:10.1039/d3ra05560d)
Supplement: RA-014-D3RA05560D-s001 [file RA-014-D3RA05560D-s001.pdf]

Electronic Supplementary Material (ESI) for RSC Advances.
This journal is © The Royal Society of Chemistry 2023

Supplementary file

Iron oxide/Graphene oxide nanocomposite synthesis using atmospheric cold plasma

Andjelika Bjelajac,*[a] Adrian-Marie Phillipe, [a] Jérôme Guillot, [a] Jean-Baptiste Chemin, [a] Patrick Choquet, [a] and Simon Bulou [a]

[a] *Luxembourg Institute of Science and Technology, 28, avenue des Hauts-Fourneaux, L-4365 Esch-sur-Alzette, Luxembourg*

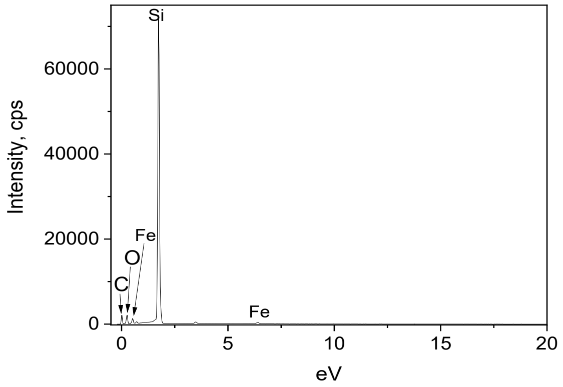

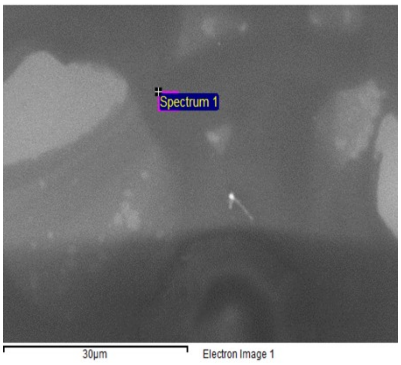

Fig. S1 EDX spectrum of the deposit obtained with plasma, using ethanol as a solvent for Fe precursor
